# Validation of the short version of the 10/66 dementia diagnosis in multiethnic Asian older adults in Singapore

**DOI:** 10.1186/s12877-017-0475-7

**Published:** 2017-04-21

**Authors:** Edimansyah Abdin, Janhavi Ajit Vaingankar, Louisa Picco, Boon Yiang Chua, Martin Prince, Siow Ann Chong, Mythily Subramaniam

**Affiliations:** 1 Research Division, Institute of Mental Health, Singapore, 539747 Singapore; 2 Health Service and Population Research Department, Institute of Psychiatry, Psychology & Neuroscience, King’s College London, London, UK

**Keywords:** 10/66 dementia, Validity, Concurrent validity, Older adults, Dementia, Asian

## Abstract

**Background:**

To validate the short version of the 10/66 dementia diagnosis against the standard version of the 10/66 dementia diagnosis and clinical diagnosis and examine concurrent validity with the World Health Organisation Disability Assessment schedule and care needs in a multiethnic Asian older adult population in Singapore.

**Methods:**

Data from the Well-being of the Singapore Elderly study, a nationally representative survey of the older Singapore Resident population aged 60 years and above was used. The validity of the short version of the 10/66 dementia diagnostic criteria derived from the Community Screening Instrument for Dementia, the modified Consortium to Establish a Registry of Alzheimer’s Disease 10-word list delayed recall and the EURO-D depression screen were examined against the standard version of the 10/66 dementia diagnosis and clinician diagnosis as a gold standard. Concurrent validity was tested by examining the relationships between the short version 10/66 dementia diagnosis, disability and care needs.

**Results:**

A total of 2373 respondents who had completed data on the short version diagnosis were included in this study. The majority (82.63%) of respondents were of Chinese descent, 9.86% were Malays, 6.12% were of Indian descent and 1.39% belonged to other ethnic group. We found the short version 10/66 dementia diagnosis showed almost perfect agreement with the standard version 10/66 dementia diagnosis (kappa = 0.90, AUC = 0.96) and substantial agreement with clinical diagnosis (kappa = 0.70, AUC = 0.87). The weighted prevalence of dementia in the population was slightly higher based on the short version diagnosis than the standard version diagnosis (10.74% vs. 10.04%). We also found that those with the short version 10/66 dementia were significantly associated with higher disability (β = 28.90, 95% CI = 23.62, 9.62) and needed care occasionally (OR =35.21, 95% CI = 18.08, 68.59) or much of the time (OR = 9.02, 95% CI = 5.21, 15.61).

**Conclusions:**

The study found that the short version 10/66 dementia diagnosis has excellent validity to diagnose dementia in a multiethnic Asian population in Singapore. Further research is required to determine the usefulness of this diagnosis in clinical practice or institutional settings to aid early detection and intervention for dementia.

## Background

Dementia can be defined as "a clinical syndrome caused by neurodegeneration, characterized by progressive deterioration in cognition in domains such as memory, learning, orientation, language, and judgment" [1]. Dementia has increasingly become an important cause of disease burden among older adults around the globe. According to Alzheimer’s Disease International (ADI), the number of people living with dementia worldwide was estimated to be 44.3 million in 2013, and is estimated to reach 75.6 million by 2030 and 135.5 million by 2050 [1]. For the Asian region, the prevalence of dementia is projected to increase from 4.3 million new cases per year in 2005 to 19.7 million new cases by 2050 [2]. It is estimated that the total cost associated with dementia is US$185 billion [3]. In Singapore, the the number of people with dementia was estimated to be 45,000 in 2015 which is estimated to reach 103,000 in 2030 and 241,000 by 2050 [3]. A recent study in Singapore reported that the prevalence of dementia was 10% and identified that older age, lower education, homemaker and retired status, and a history of stroke diagnosis were associated with a higher risk of dementia [4]. It is well established that dementia is a devastating disorder for those who experience it, for their caregivers, their families and society as a whole [5]. Previous studies have shown that dementia is a leading contributor to disability and care need among older people, resulting in increased cost to society [6–9].

There has been an increase in epidemiological research related to dementia, with attempts to harmonise the studies across geographical areas, using common methods and diagnostic criteria [10]. The standard version of the 10/66 dementia diagnostic criteria developed by the 10/66 Dementia Research Group (https://www.alz.co.uk/1066/) has been used widely to ascertain and compare dementia prevalence among older people worldwide [8, 11, 12]. Its diagnostic algorithm uses predicted probabilities derived from logistic regression coefficients incorporating data from the (i) Community Screening Instrument for Dementia (CSI-D) [13] administered to participants (32 items) and informants (26 items) to produce a cognitive score (CSI-D COGSCORE) and an informant score (CSI-D RELSCORE), (ii) Geriatric Mental State-Automated Geriatric Examination for Computer Assisted Taxonomy (GMS-AGECAT) diagnosis (157 items) [14], and (iii) modified Consortium to Establish a Registry of Alzheimer’s Disease (CERAD) [15] ten-word list learning tasks with delayed recall. The standard 10/66 dementia diagnostic criteria were translated into languages used in the developing countries including Chinese and Tamil. The standard 10/66 dementia diagnosis algorithm has been cross-culturally validated against Diagnostic and Statistical Manual, Fourth Edition (DSM-4) dementia diagnosis by clinicians in 25 centres in pilot studies worldwide [11] including different ethnicities and language groups in India, China, countries in Southeast Asia, Latin America and the Caribbean, as well as Africa. It was found to have a high sensitivity (94%) with low false positive rates in those with depression (15%), higher education (3%) and lower education (6%). It has been reported that the discriminatory ability of the standard 10/66 dementia diagnosis was best in China, southeast Asia and India and worst, although still perfectly acceptable, in Latin America [11]. The standard 10/66 dementia diagnosis has also shown excellent discriminatory ability to diagnose dementia in an Arabic speaking older population in Lebanon [16] and in Asian older adults in Singapore [4].

Recently, the short version of the 10/66 dementia diagnostic criteria has been developed for epidemiological studies of dementia where Geriatric Mental State (GMS) interview is not feasible using existing data from the 10/66 pilot studies (*n* = 2885) and were further validated using data from the first wave of 10/66 surveys carried out at 12 urban and rural sites in eight countries (Cuba, Dominican Republic, Peru, Mexico, Venezuela, India, China and Puerto Rico) [11, 17]. Its diagnostic algorithm uses predicted probabilities derived from the logistic regression coefficient incorporating data from the CSI-D COSGSCORE, CSI-D RELSCORE, the modified CERAD ten-word list learning with delayed recall and the European Depression scale (EURO-D) instead of the GMS-AGECAT diagnostic output to produce dementia diagnosis. The EURO-D is a 12-item depression screening scale that can be administered briefly within 3–5 min [17]. The development of the short version was initiated as an alternative instrument for the standard version of the 10/66 diagnostic criteria to reduce administration time by the GMS-AGECAT that could enhance the efficiency of administration of the 10/66 diagnostic assessment especially in research in institutional settings or in clinical practice. The average amount of time spent by trained interviewers to administer the standard version of the 10/66 diagnostic assessment with the GMS-AGECAT can last between 2 and 3 h. The short version has demonstrated high sensitivity (94.2%) with a specificity of 80.2% in people with depression, 96.6% in the high-education group and 92.7% in the low-education group [17].

Singapore is a country in Southeast Asia with a resident population of 3.9 million. It has one of the world’s fastest aging multi-ethnic population of which 74.3% are Chinese, 13.3% are Malays, 9.1% are Indians, and 3.2% belong to other ethnicities [18]. Given the potential of the short version of 10/66 dementia diagnosis in identifying dementia in sites and situations where time is more limited for interviewer training and/or dementia assessment, it is thus important to investigate its validity in various populations [17]. The present study aims to validate the short version of the 10/66 dementia diagnosis against the standard version of the 10/66 dementia diagnosis and clinical diagnosis and examine concurrent validity of the short version with the World Health Organisation Disability Assessment schedule (WHODAS II) and care needs in a multiethnic Asian older adult population in Singapore using population-based dataset from the Well-being of the Singapore Elderly (WiSE) study.

## Methods

The WiSE study was a comprehensive single phase, cross-sectional survey conducted in the year 2013 to determine the prevalence of dementia among older adults (aged 60 years and above) in Singapore. The study has been described in further detail previously [4]. Inclusion criteria for the study comprised Singapore residents (including Singapore citizens and permanent residents) aged 60 years and above who were living in Singapore at the time of the survey. Respondents who were in day care centres, nursing homes, and institutions were also included. Respondents were randomly selected using a disproportionate stratified sample design via a national registry that maintains the names and socio-demographic details such as age, gender, ethnicity and addresses of all residents in Singapore. The study also included an informant who was defined as the person who knew the older adult best. If respondents were unable to answer the questions, informants were then asked the questions to maximise data capture. The informant was also further interviewed using the Informant Questionnaire [19] which covered information relating to the sociodemographic background of the informant, the care needs of the older respondents, the informant’s observations of cognitive and functional decline and the informant rated presence and severity of any behavioural and psychological symptoms measured by the Neuropsychiatry Inventory Questionnaire [20]. The sample size was derived from a statistical power calculation for binary proportions after adjusting for design effect using the established prevalence rate of dementia in Singapore, with precision of 5%. We found the margin of error (ME) using a dementia prevalence of 5.2% was between 1.5% and 3%. The ME for the strata defined by age and ethnic groups was 1.0 to 3.5%, while the relative standard error was below 30%. These estimates showed that our target sample size of 2500 was estimated to be adequate to provide sufficient precision to measure the prevalence of dementia [4]. All respondents and informants provided written informed consent and in the case of respondents who were unable to provide informed consent, written informed consent was taken from their legally acceptable representative/next of kin.

Second-level assessments conducted by experienced independent clinicians as a gold standard for dementia diagnosis were included in the current study. Comparisons with second-level assessments were designed to determine whether diagnostic classifications based on the short version of the 10/66 dementia diagnosis were different from blinded clinical diagnosis by experienced clinicians who underwent training sessions to standardize the assessment and diagnosis based on the DSM-IV criteria for dementia. Out of 258 respondents with and without dementia diagnosis based on the 10/66 dementia diagnosis who were randomly selected and invited to participate in the second level assessment, 133 participants who agreed to the second level assessment were included in current analysis. The study was approved by the relevant institutional ethics review boards (National Healthcare Group Domain Specific Review Board and the SingHealth Centralised Institutional Review Board).

### Measures

The following instruments were included in current analysis:

- The EURO-D is a 12-item instrument (pertaining to depressed mood, pessimism, wishing death, guilt, sleep, interest, irritability, appetite, fatigue, concentration, enjoyment and tearfulness), with the items extracted from the Geriatric Mental State examination [21, 22]. Each item is scored 0 (symptom not present) or 1 (symptom present) to generate a simple ordinal scale with a maximum score of 12.
- The CSI-D [13] is a community screening interview for dementia (incorporating the CERAD Animal Naming Verbal Fluency Task) which incorporates elements of memory, orientation, naming, language expression and comprehension. The interview can be administered to participants (32 items) and informants (26 items) to generate cognitive score (CSI COGSCORE) based on an item weighted total score from each participant interview based on the number of correct answers (0 = cognitively impaired, 32 = no cognitive impairment) and an informant score (CSI-D RELSCORE) based on unweighted total score from the informant interview [23].
- The modified CERAD ten-word list learning task with delayed recall was extracted from the adapted CERAD ten-word learning task that requires participants to recall the ten words (i.e. butter, arm, letter, queen, ticket, grass, corner, stone, book and stick) that they remember, giving a total learning score out of 10 [15].
- GMS [24] is a semi-structured interview which can be administered between 25 and 40 min [11]. It applies Geriatric Mental State-Automated Geriatric Examination for Computer Assisted Taxonomy (GMS-AGECAT) a computer algorithm to produce dementia diagnosis. Briefly, GMS-AGECAT divides 157 symptom components into four syndrome clusters in Stage 1 diagnosis: organicity (dementia); schizophrenia and related paranoia; depression; and anxiety neurosis. A severity level is provided for each syndrome, ranging from 0 (no symptoms) to 5 (very severely affected). Level three and greater constitute a ‘case’ while levels one and two represent ‘subcases’. Stage 1 diagnosis subsequently is organised into a stage two diagnosis on the basis of precedence determined by a hierarchical algorithm.
- The 12-item interviewer-administered version of the World Health Organisation Disability Assessment schedule (WHODAS II) was used to assess disability in the study. For each item, individuals had to estimate the difficulties due to health problems during the previous 30 days from none = 1 to extreme/cannot do = 5. The total score of the 12 items was obtained where higher scores indicated high disability.
- Care need was ascertained by the interviewer asking the informant a series of open ended questions that included the following: Who shares the home with the respondent? What kind of help does the respondent need inside of the home and outside of the home? Who, in the family, is available to care for the respondent? What help do you provide? Do you help to organise care and support for the respondent? Is there anyone else in the family who is more involved in helping than you? What about friends and neighbours? Based on the responses provided by the informant, the interviewer then coded whether the respondent needed care much of the time, occasionally or not at all.

The 10/66 questionnaires were available in English, Chinese and Tamil but not in Malay. During the pilot phase of the study, a significant number of respondents were identified who could only speak dialects, i.e., Hokkien, Cantonese, or Teochew. The questionnaires were subsequently translated into Malay and these three major dialects. The translation procedure was conducted according to the guidelines outlined by the WHO. A translation was derived from two independent forward translations. An expert panel comprising professional translators, content experts and a layperson was convened to identify and resolve any inadequate expressions in the translation and identify discrepancies between the translated and original version. Cognitive interviews and pre-testing were conducted with individuals representing the target population to assess whether the translated version was understood in the manner intended. Socio-demographic information obtained included age at interview (60–74 years, 75–84 years, 85 years and above), gender, ethnicity (Chinese, Malay, Indian, and other), marital status (never married, married/co-habiting, divorced or separated and widowed), educational level (primary and below, secondary, and tertiary) and employment status (employed, homemaker, retired, and unemployed) [4].

### Statistical analysis

Statistical analyses were carried out using the SAS software version 9.2 (SAS Institute Inc., Cary, NC, USA). Descriptive statistics were performed to establish the sociodemographic characteristics and dementia diagnosis. The estimates were weighted to adjust for oversampling and non-response and post-stratified for age and ethnicity distributions between the survey sample and the Singapore elderly population in 2011 to ensure that the survey findings were representative of the Singapore elderly population. This approach has been recommended when analysing survey data to take into account the complex sample design and weighting [25]. The 10/66 dementia diagnosis according to the short version diagnostic algorithm developed by Stewart et al. [17] and standard version diagnostic algorithm of the 10/66 dementia diagnosis developed by Prince et al. [11] based on cut-point predicted probabilities of more than 0.20 and 0.25 derived from the logistic regression coefficients were applied to all participants. Using the cut-off defined above, we estimated the sensitivity, specificity, false positive value (FPV), positive predictive value (PPV), false negative value (FNV), Cohen’s kappa and area under the receiver operative characteristics curve (AUC) of the short version against the standard version of the 10/66 dementia diagnosis and clinician diagnosis. Kappa was used because it is a commonly used measure of diagnostic concordance. However, due to its disadvantage of varying across populations that differ in prevalence even when the populations do not differ in sensitivity and specificity, we have supplemented it with AUC which is insensitive to prevalence, and equals (sensitivity + specificity)/2 [26]. Prevalence of dementia across sociodemographic characteristics of the sample according to the short version and standard version diagnostic algorithms were also estimated. For each demographic category, we evaluated biased prevalence estimates by calculating the difference in prevalence across short version and standard version diagnostic algorithms and obtained their AUC and Kappa agreement statistics. The concurrent validity of the short version of the 10/66 dementia diagnosis with other measures such as WHODAS II disability scores and care needs were examined using multiple linear regression and multinomial logistic regression analyses after controlling for sociodemographic variables. Statistical significance was evaluated at the 0.05 level using 2-sided tests.

## Results

### Sociodemographic characteristics

Two thousand five hundred sixty-five respondents were recruited in the study giving a response rate of 65.55%. Out of the 2565 recruited, a total of 2421 respondents whose informants completed the requisite questionnaires were identified from the WiSE database, of whom, 2373 respondents had completed data on the short version dementia diagnosis criteria and were included in the analysis. The sociodemographic characteristics of the respondents are shown in Table 1. The sample comprised 57.08% female and 42.92% male respondents. The majority of the sample was aged between 60 to 74 years (75.19%), of Chinese ethnicity (82.63%), and currently married (65.29%).

**Table 1:** Socio-demographic characteristics (*n* = 2373)

	Unweighted N	Unweighted %	Weighted %
Age group (years)			
60–74	1382	58.24	75.19
75–84	616	25.96	19.14
85+	375	15.80	5.68
Gender			
Men	1014	42.73	42.92
Women	1359	57.27	57.08
Ethnicity			
Chinese	911	38.39	82.63
Malay	714	30.09	9.86
Indian	717	30.21	6.12
Others	31	1.31	1.39
Marital status			
Never married	107	4.51	6.92
Married/cohabiting	1392	58.71	65.29
Widowed	781	32.94	22.86
Divorced/separated	91	3.84	4.92
Education			
None	487	20.64	16.86
Some, but did not complete primary	569	24.12	23.88
Completed primary	590	25.01	24.29
Completed secondary	479	20.31	22.53
Completed tertiary	234	9.92	12.43
Employment status			
Paid work (part-time and full-time)	628	26.78	33.37
Unemployed	30	1.28	1.46
Homemaker	767	32.71	27.15
Retired	920	39.23	38.02

### Validity against standard version of the 10/66 diagnosis and clinical diagnosis

Table 2 shows the diagnostic validity of the short version of the 10/66 dementia diagnosis against the standard version of the 10/66 dementia and gold standard diagnosis (clinician diagnosis). We found the short version 10/66 dementia diagnosis showed almost perfect agreement with the standard version 10/66 dementia diagnosis (kappa = 0.90, AUC = 0.96). The high agreement between two algorithms also led to high proportion of standard 10/66 dementia cases being detected in the short version algoritm (sensitivity = 94.45%), with high specificity (97.64%). The sensitivity (91.11%) and specificity (82.95%) of the short version 10/66 diagnosis was high against clinical diagnosis with substantial agreement between the two diagnoses (kappa =0.70, AUC = 0.87).

**Table 2:** Validity of the short version of the 10/66 diagnosis

	Standard 10/66 diagnosis (*n* = 2373)	Clinician diagnosis (*n* = 133)
Sensitivity	94.45%	91.11%
Specificity	97.64%	82.95%
FPV	11.60%	26.79%
FNV	1.07%	5.19%
PPV	88.40%	73.21%
% agreement	97.13%	85.71%
Kappa*	0.90	0.70
AUC	0.96	0.87

Note: *Kappa values: <0 = Less than chance agreement, 0.01–0.20 = slight agreement, 0.21–0.40 = fair agreement, 0.41–0.60 = moderate agreement, 0.61–0.80 = substantial agreement, 0.81–0.99 almost perfect agreement

### Comparison of dementia prevalence across sociodemographic characteristics according to the short version and standard diagnostic algorithms

In the overall WiSE sample, we found that the weighted prevalence of dementia in the population was slightly higher based on the short version algorithm than the standard diagnostic algorithm (10.74% vs. 10.04%). When comparing the prevalence rates across sociodemographic characteristics, the short version algorithm reported higher prevalence rates of dementia than the standard algorithm across almost all socidemographic groups (difference in prevalence rates ranged from 0.34% to 3.76%) except among those of Malay ethnicity and those with primary education. The kappa agreement varied across sociodemographic characteristics and ranged from moderate (0.47) to almost perfect agreement (0.95) (Table 3). The AUC ranged from 0.90 to 1.00.

**Table 3:** Comparison of dementia prevalence across age, gender, ethnicity, marital status, education and employment groups, according to the short version and standard version diagnostic algorithms

	Short version 10/66 diagnosis		Standard version 10/66 diagnosis		Percentage difference	Kappa	AUC
	Weighted %	SE	Weighted %	SE			
Age group (years)							
60–74	4.33	0.76	2.99	0.62	1.34	0.70	0.93
75–84	22.96	2.37	21.36	2.31	1.60	0.86	0.94
85+	58.11	3.36	55.47	3.40	2.64	0.89	0.94
Gender							
Men	8.48	1.11	7.22	1.02	1.26	0.86	0.96
Women	12.81	1.08	11.19	0.95	1.62	0.84	0.95
Ethnicity							
Chinese	11.42	0.90	9.74	0.80	1.68	0.84	0.95
Malay	9.68	0.95	9.85	0.95	−0.17	0.95	0.97
Indian	9.20	0.91	7.61	0.78	1.59	0.82	0.95
Others							
Marital status							
Never married	8.82	3.38	5.33	2.53	3.49	0.71	0.97
Married/cohabiting	6.31	0.82	5.52	0.76	0.79	0.81	0.93
Widowed	25.05	2.07	22.57	1.89	2.48	0.88	0.96
Divorced/separated	6.51	3.33	3.61	2.45	2.90	0.70	0.98
Education							
None	24.82	2.62	21.59	2.37	3.23	0.84	0.94
Some, but did not complete primary	14.23	1.91	10.47	1.59	3.76	0.81	0.97
Completed primary	8.10	1.51	9.16	1.59	−1.06	0.85	0.90
Completed secondary	4.54	1.06	3.47	0.86	1.07	0.84	0.99
Completed tertiary	2.65	1.08	2.31	1.04	0.34	0.93	1.00
Employment status							
Paid work (part-time and full-time)	0.69	0.36	0.22	0.09	0.47	0.47	1.00
Unemployed							
Homemaker	13.56	1.61	11.92	1.45	1.64	0.89	0.97
Retired	17.87	1.61	15.38	1.47	2.49	0.81	0.93

### Concurrent validity with other measures

Table 4 shows the concurrent validity between the short version 10/66 dementia diagnosis and other measures. The short version 10/66 dementia diagnosis significantly and positively correlated with WHODAS II score and care needs expressed by caregivers. After adjustment for all socio-demographic variables in the multivariate analyses those with the short form 10/66 dementia were significantly associated with higher WHODAS II score (β = 28.90, 95% CI = 23.62, 9.62) and needed care occasionally (OR =35.21, 95% CI = 18.08, 68.59) or much of the time (OR = 9.02, 95% CI = 5.21, 15.61).

**Table 4:** Concurrent validity of the short version 10/66 diagnosis

	Short version 10/66 diagnosis			
	Yes	No		
	Mean (SE)	Mean (SE)	β (95% CI)	*p* value*
WHODAS II Disability	44.38 (2.3)	6.49 (0.34)	28.90 (23.62,9.62)	<0.001
	% (SE)	% (SE)	OR (95% CI)	*p* value
Need for care				
Needs care much of the time	46.27 (3.72)	2.46 (0.42)	35.21 (18.08,68.59)	<0.001
Needs care occasionally	31.46 (3.52)	6.64 (0.68)	9.02 (5.21,15.61)	<0.001
Does not need care	22.27 (3.56)	90.89 (0.79)	Reference	

*Significant level was estimated using multiple linear and multinomial logistic regression analyses after controlling for socidemographic variables

## Discussion

The study found that the short version 10/66 dementia diagnosis algorithm has excellent validity to diagnose dementia in a multi-ethnic Asian population in Singapore. Almost perfect agreement (kappa = 0.90, AUC = 0.96) was found between diagnoses of dementia based on the short version and standard version and a substantial agreement was found with clinical diagnosis (kappa = 0.70, AUC = 0.87). Our findings are consistent with a previous study [17] suggesting that the short version is a valid alternative diagnostic instrument to establish the prevalence of dementia in the Asian population. It can potentially be used where training interviewers for administration of long or semi-structured diagnostic interviews such as the GMS may not be feasible, and /or in routine practice or assessments where there is insufficient interview time for administering these instruments. However, it should be noted that the use of the EURO-D alone rather than the full GMS instrument in future studies would result in researchers being unable to establish the prevalence of ICD-10 and DSM-IV depression, as well as cases and subsyndromal cases of anxiety and psychosis in their studies.

The short version generally over-estimated dementia prevalence compared to the standard version of the 10/66. The difference in the prevalence estimates across sociodemographic charactersitics by the short version as compared the standard version ranged from 0.34% to 3.76%. The difference in the prevalence estimate did not vary substantially suggesting that it should be acceptable for population-based research and suited for studies designed to make comparisons across sociodemographic characteristics. We found the agreement and AUC between the two diagnoses stratified by sociodemographic characteristics to be consistently higher. There was low kappa agreement between these two diagnoses among those belonging to the ‘paid work’ subsample of employment category. The low value was expected due to lower number of dementia cases detected by both these two diagnoses. Several studies [26, 27] have criticized the kappa statistic for being inherently dependent on prevalence and claimed that this dependency introduces bias and statistical artefacts to estimates of accuracy. In this case, agreement between these two diagnoses should be based on AUC value [26, 28] which indicates perfect agreement between the two diagnoses.

The study also found support for the concurrent validity of the short version 10/66 diagnosis. The short version of the 10/66 diagnosis positively correlated with WHODAS II scores and care needs. Those diagnosed with the short version of the 10/66 dementia diagnosis reported higher disability and needed more care than those without dementia. Studies that have used the standard version of the 10/66 diagnosis have similarly found a significant positive association with disability [4, 8] and care needs [29, 30]. For example, Llibre Rodriguez et al. [8] and Subramaniam et al. [4] found that those diagnosed with the standard 10/66 dementia diagnosis but not confirmed by the DSM-IV criterion were consistently disabled compared with those who did not meet the 10/66 criteria.

There are some limitations to the current study. This was a secondary analysis of data from a cross-sectional study; and the second level assessment by clinicians was not specifically designed to validate the short version of the 10/66 diagnosis. The EURO-D was administered nested within the full GMS and scores and diagnostic validity may have been different if it was administered on its own. Given the overestimation of dementia prevalence by the short version 10/66 diagnosis, there is a possibility of the short version producing slightly higher false positive cases if the short version is used in population-based surveys and clinical settings. Our study has several strengths including a large and multi-ethnic sample and the use of structured instruments to measure dementia. It is one of the few studies that have validated the short version of the 10/66 diagnosis in a representative sample. With regards to clinical implications, the current findings support the evidence that the short version 10/66 dementia diagnosis can be established by using 12 items from the EURO-D depression with 3–5 min administration time instead of 157 items from the GMS-AGECAT instruments with 25–40 min administration time. Hence, with smaller number of items used, the short version of the 10/66 dementia diagnosis may be useful as a routine diagnostic tool to identify older adults with dementia in a clinical setting or population health monitoring survey across ethnic groups in Singapore. Furthermore, given that our findings have shown that the short version of the 10/66 dementia diagnosis is associated with substantial impairment in functioning as measured by WHODAS II disability scores and higher dependency among older adults as reported by higher care need expressed by caregivers, identification and treatment of dementia among elderly could result in a better prognosis which can facilitate treatment planning.

## Conclusions

In conclusion, this study has demonstrated that the short version of the 10/66 dementia diagnosis is a valid alternative diagnostic instrument to establish the prevalence of dementia in an Asian population. However further research is required to determine the usefulness of this diagnosis in clinical practice or institutional settings to aid early detection and interventions for dementia.

## Abbreviations

CERAD: Consortium to Establish a Registry of Alzheimer’s Disease (CERAD)
CSI-D: Community Screening Instrument for Dementia
DSM-4: Diagnostic and Statistical Manual, Fourth Edition
EURO-D: European Depression Scale
GMS-AGECAT: Geriatric Mental State-Automated Geriatric Examination for Computer Assisted Taxonomy
WHODAS: World Health Organisation Disability Assessment schedule
